# Structural Features Predict Sexual Trauma and Interpersonal Problems in Borderline Personality Disorder but Not in Controls: A Multi-Voxel Pattern Analysis

**DOI:** 10.3389/fnhum.2022.773593

**Published:** 2022-02-23

**Authors:** Harold Dadomo, Gerardo Salvato, Gaia Lapomarda, Zafer Ciftci, Irene Messina, Alessandro Grecucci

**Affiliations:** ^1^Unit of Neuroscience, Department of Medicine and Surgery, University of Parma, Parma, Italy; ^2^Brain and Behavioral Sciences, University of Pavia, Pavia, Italy; ^3^Milan Center for Neuroscience, School of Medicine and Surgery, University of Milano Bicocca, Milan, Italy; ^4^Cognitive Neuropsychology Centre, ASST Grande Ospedale Metropolitano Niguarda, Milan, Italy; ^5^Clinical and Affective Neuroscience Lab – Cli.A.N. Lab, Department of Psychology and Cognitive Science, University of Trento, Rovereto, Italy; ^6^Mercatorum University, Rome, Italy; ^7^Centre for Medical Sciences, CISMed, University of Trento, Trento, Italy

**Keywords:** multi-voxel pattern analysis, borderline personality disorder, multiple kernel learning, machine learning, child trauma, biomarkers, brain imaging

## Abstract

Child trauma plays an important role in the etiology of Bordeline Personality Disorder (BPD). Of all traumas, sexual trauma is the most common, severe and most associated with receiving a BPD diagnosis when adult. Etiologic models posit sexual abuse as a prognostic factor in BPD. Here we apply machine learning using Multiple Kernel Regression to the Magnetic Resonance Structural Images of 20 BPD and 13 healthy control (HC) to see whether their brain predicts five sources of traumas: sex abuse, emotion neglect, emotional abuse, physical neglect, physical abuse (Child Trauma Questionnaire; CTQ). We also applied the same analysis to predict symptom severity in five domains: affective, cognitive, impulsivity, interpersonal (Zanarini Rating Scale for Borderline Personality Disorder; Zan-BPD) for BPD patients only. Results indicate that CTQ sexual trauma is predicted by a set of areas including the amygdala, the Heschl area, the Caudate, the Putamen, and portions of the Cerebellum in BPD patients only. Importantly, interpersonal problems only in BPD patients were predicted by a set of areas including temporal lobe and cerebellar regions. Notably, sexual trauma and interpersonal problems were not predicted by structural features in matched healthy controls. This finding may help elucidate the brain circuit affected by traumatic experiences and connected with interpersonal problems BPD suffer from.

## Introduction

The Borderline Personality Disorder (BPD) is a complex mental disorder with a characteristic pervasive pattern of instability on affect regulation showing different dysphoric states shifting from one interpersonally reactive mood to another with great fluidity (Zanarini et al., 1998; Stiglmayr et al., 2001; Dadomo et al., 2016, 2018). Impulsiveness (Mortensen et al., 2010; Lapomarda et al., 2021a,b), strong feelings of very deep inadequacy, dissociative experiences (Zanarini et al., 1990; Koenigsberg et al., 2002), interpersonal relationships and problem with self-image (Lieb et al., 2004; De Panfilis et al., 2019)are the main characteristics of BPD. This disorder affects approximately 1–3% of the general population (Lenzenweger et al., 2007; Trull et al., 2010) up to 10% of outpatient psychiatric patients (Zimmerman et al., 2005) 20% of hospitalized patients and 15–25% of the clinical population (McGlashan et al., 2000).

Various types of adverse life events in childhood, including experiences of neglect and abuse, would appear to be one of the most important factors (Zanarini et al., 1989; Lobbestael et al., 2010). The most frequent of these is childhood sexual abuse, reported by 40–71% of patients linked to the severity of the abuse itself (Shearer et al., 1990; Paris et al., 1994; Zanarini et al., 2002). Consistent evidence show as sexual abuse during childhood is a reliable predictor of chronic PTSD (Müller et al., 2018), is strongly linked to ultra-high risk of psychosis (UHR), first –episode psychosis (FEP; Ciocca et al., 2021). Sexual trauma is particularly relevant for the development of addiction (Poppa et al., 2019) and it is often associated with the ineffectiveness of pharmacological treatment of anxiety disorders (Kim et al., 2021).

Among several traumatic life events, childhood sexual abuse and emotional maltreatment seem to constitute a keys etiological risk factor for the BPD (Johnson et al., 1999; Zlotnick et al., 2003; Lobbestael and Arntz, 2010; Preißler et al., 2010; Dadomo et al., 2016, 2018; de Aquino Ferreira et al., 2018). These traumatic events have a specific effect on the subject’s behavior and accurately predicts the symptom class observed in the borderline patients such as affective and interpersonal dysfunctionalities, which negatively impact on their relationships (Ball and Links, 2009). Indeed, BPD patients are characterized by impaired mental state attribution, impairment in cognitive empathy and in emotion recognition abilities (Preißler et al., 2010; De Panfilis et al., 2019).

Numerous neuroimaging studies have explored BPD features in recent years, leading to the identification of some cerebral structural and functional alterations associated with the pathogenesis of BPD. Up to 2013, the majority of studies indicated that structural differences in the amygdala hub, hippocampus and cingulate cortex are involved in affective deficits (Minzenberg et al., 2008; Nunes et al., 2009; Ruocco et al., 2012; Piretti et al., 2020). More recently, alterations in frontal (e.g., orbifrontal cortex, medial prefrontal cortex; Aguilar-Ortiz et al., 2018), cortical and subcortical regions (Ruocco et al., 2016; Stanley et al., 2018; Davies et al., 2020; Lapomarda et al., 2021a,b) have also been identified. Yet, if a meta-analysis confirmed this constellation of brain regions (Yu et al., 2019), other extended abnormality in temporal cortex and cerebellum (Schulze et al., 2016) will complete the puzzling picture. Therefore, a potential circuit involved in BPD seems still far from been exhaustive.

One of the main limitations of the previous neuroimaging studies on BPD concerns the use of mass univariate methods to compare groups (e.g., Minzenberg et al., 2008; Grecucci et al., 2015; Aguilar-Ortiz et al., 2018; Sorella et al., 2019; Pappaianni et al., 2020; Lapomarda et al., 2021a). Typically, comparisons of mean imaging indices between patients and healthy controls, across different brain regions using region of interest (ROI) or voxel-based techniques have been performed. This approach clearly has pro et contra: morphometric approaches, such as Voxel-based Morphometry (VBM), allow evaluating between-groups differences in certain brain structures, as a univariate technique it directly compares different voxels in different individuals’ brains, neglecting their interrelationships. Furthermore, VBM sensitivity from large cortical areas to smaller subcortical structures is dramatically reduced (Aguilar-Ortiz et al., 2018). Therefore, it is clear that the high variability of the previous results is probably related to methodological differences and limitations, which in turn influenced the results of the various meta-analyzes. In this background, the use of multivariate methods would instead provide detailed information on how the regions are correlated, identifying naturally grouped circuits (Grecucci et al., 2016; Sorella et al., 2019; Saviola et al., 2020; Lapomarda et al., 2021a,b).

Pattern recognition methods, such as multi-voxel pattern analysis (MVPA), are inherently multivariate and use information distributed over multiple voxels as well as being sensitive to spatially distributed effects (Norman et al., 2006). Of note, MVPA can be used to predict ongoing psychological variables such as symptom severity or psychological variables (Davies et al., 2020). Multiple Kernel Regression (MKR), is a pattern recognition algorithm used in MVPA, a sparse machine learning method that can be used for the identification of the most relevant sources, such as psychological variables based on anatomical location (Mourao-Miranda et al., 2012). It can also help determine which regions of the brain contribute most to explaining psychological variables. In this regard, MVPA has recently been applied to patients with various psychiatric disorders (Orrù et al., 2012) or to investigate brain changes associated with clinical improvement (Whitfield-Gabrieli et al., 2016; Takamiya et al., 2020).

In this study, we aim at applying multivariate methods, using MVPA based on MKR, to explore brain circuits that predict trauma and symptoms severity. To do this we will use two tools: the Child Trauma Questionnaire (CTQ) and the Zanarini Rating Scale for Borderline Personality Disorder (Zan-BPD). The CTQ is a self-assessment tool used to evaluate the traumatic experiences experienced during childhood. On the other hand, the Zan-BPD measures the severity of symptoms of an affective nature such as anger, feelings of emptiness and mood instability; cognitive such as identity disturbance disassociation and paranoia; symptoms related to impulsivity such as self-mutilative/suicidal efforts and finally interpersonal symptoms such as intense, unstable relationships and frantic efforts to avoid abandonment of the borderline patient.

Combining the clinical scales and the application of whole-brain MVPA based on MKR in BPD patients, we sought to test two hypotheses. The first hypothesis is that sexual trauma and more specifically sexual abuse being the main etiologic factor in BPD can be successfully predicted by brain features. *Inter alia* we expect that basal ganglia and Heschl’s gyrus is part of this circuit predicting both Child trauma (Zhang et al., 2015; Quidé et al., 2017) and symptomatology of borderline patients. The second hypothesis is that brain features also predict interpersonal problems, one of the main features of BPD patients. We predict that structural alterations in temporal cortex will be predictive of interpersonal problems in BPD measured by the Zan-BPD questionnaire.

## Materials and Methods

### Participants

Twenty patients with borderline personality disorder (BPD, *M*_*age*_ = 35.75, *SD*_*age*_ = 8.61), and 13 healthy participants as controls (HC, *M*_*age*_ = 32.53, *SD*_*age*_ = 8.3), matched for age (p = 0.63) and sex (p = 0.48) were taken into consideration Age and gender differences were assessed *via t*-test. Note that, three controls were excluded because they did not fill the CTQ questionnaire. All the data were extracted from the Clinical Research Imaging Centre in Edinburgh (OpenNeuro database, accession number ds000214) (Poldrack and Gorgolewski, 2017). The recruitment took place in outpatient and support services from around Edinburgh.

**FIGURE 1:**
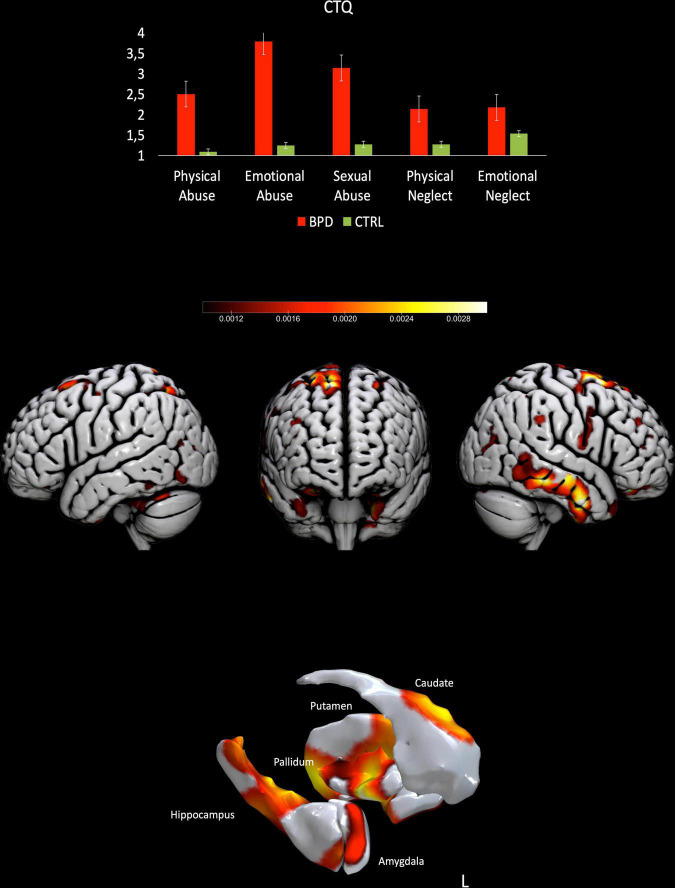
Results from the prediction of sexual trauma for BPD patients. Upper part, results from the CTQ-subscales scores for both BPD patients and controls. Lower part, surface plots, including subcortical reconstruction of the significant regions predicting Sexual trauma in BPD patients.

The exclusion criteria were the presence of neurological disease, or mental illness rather than BPD (SCID-II, SCID-IV), and the use of psychoactive substance, pregnancy, MRI contraindications. The BPD diagnosis was verified using Structured Clinical Interview for DSM-IV (SCID-II). The CTQ was administered to both patients and controls, although three control subjects did not fill the questionnaire. Zanarini Rating Scale for Borderline Personality Disorder (ZAN-BPD) was administered to assess the current symptoms only to BPD. See Figure 1. Demographic information about participants are displayed in Table 1. A high-resolution T1-weighted 3D magnetization prepared rapid gradient echo (MPRAGE) scan was acquired for each participant *via* 3T Siemens Magneton (Verio) MRI scanner with TR = 2300 ms, TE = 2.98, 160 slices.

**TABLE 1:** Demographic information about participants. Values in round brackets are the standard deviations.

	DEMOGRAPHIC INFORMATION		
	BPD	HC	*p*-values
**Participants**	20	13	
**Age (yrs)**	36.75 (±8.61)	32.53 (+8.3)	*p* = 0.63
**Gender**	*F* = 17	*F* = 11	*p* = 0.48
**Education**	≥8	≥8	
**Screening**	Neurological disease, psychoactive substance, mental illness (SCID-II, SCID-IV)	Neurological disease, psychoactive substance, mental illness (SCID-II, SCID-IV)	
**Exclusion criteria**	Diagnosis in at least two different psychiatric categories, pregnancy, MRI contraindications, neurological disease	Diagnosis in one diagnostic category, pregnancy, MRI contraindications, neurological disease	

### Preprocessing

After quality check of the images to exclude artifacts, all data were preprocessed using the segmentation routines provided by the Computational Anatomy Toolbox (CAT12)^1^, a toolbox available for SPM12 software^2^ in the MATLAB environment. Segmentation of gray and white matter, and cerebrospinal fluid was thus obtained. Modulated normalized writing option was chosen. Diffeomorphic Anatomical Registration through Exponential Lie algebra (DARTEL) tools, a potential alternative to SPM’s traditional registration approaches that operates using a whole-brain approach, was used (Yassa and Stark, 2009; Grecucci et al., 2016; Pappaianni et al., 2018). Normalization to MNI space with spatial smoothing [full-width at half maximum of Gaussian smoothing kernel (8)] was then applied on DARTEL images.

### Data Analysis

Machine learning based on MKR method was carried out in the Pattern Recognition for Neuroimaging Toolbox (PRoNTo) (Schrouff et al., 2013, 2018) and Matlab scripts. BPD and HC were analysed separately to predict the psychological variables (questionnaires scores). Multiple Kernel Learning (MKL; Schrouff et al., 2014) simultaneously learns the contribution of each brain region, previously defined by an atlas, to the decision. Thus, MKL lead to improved generalization performance and identifies a subset of relevant brain regions for the predictive model. To do this MKL combines the information coming from each voxel of different brain regions. To avoid computational complexity, kernels, or similarity matrices, are computed to reduce the input space in a few dimensions. Different brain areas correspond to a different kernel. After weights estimation, every region is ordered according to its contribution to the model; thus, it can be defined as a hierarchical model (regions contributing more vs. regions contributing less). Whole brain analyses were performed using a general brain mask provided inside PRoNTo. Age and gender were regressed out to avoid confoundings. The procedure was split into a training and a testing phase. The predictive function was calculated during the training phase where the algorithm learns to predict the psychological variables of interest (CTQ scores, etc.) from structural data. Whereas, during the test phase, the algorithm was used to predict the outcome in an independent dataset. To avoid splitting the data in a training and in a test set, thus reducing the number of subjects available for each calculation, leave one subject out cross-validation was performed. In this method, the total number of subjects minus one is used for the training phase. Then the performance is assessed by predicting the excluded subject. This is iteratively repeated for every subject, so that every subject has been used for training and testing the model in the end. Then the average performance is calculated across all the testing performances. The hyperparameters were set to 0.0001 0.01 1 10 100 1000. The parameter with the highest performance (balanced accuracy, BA) is then applied to assess the model (Schrouff et al., 2013). Statistical significance of the classifications was tested using permutation testing with 1500 permutations with random assignment of group class to input image. The resulting null-hypothesis distribution was used to calculate the *p*-value of the accuracies, or the proportion of permutations that yielded a greater accuracy than the accuracy found for the classification models. The Automated Anatomical Labeling (Tzourio-Mazoyer et al., 2002) atlas, built using the WFU- Pickup Atlas toolbox of SPM and consisting of 116 brain regions was used to explore regional contribution of each classification model. Being MKR approach a hierarchical model of the brain, it was possible to derive weights contribution of each region to the decision function. Regions were ranked according to their contribution to the model and averaged across folds. Only regions with >1% contribution to the decision function f are displayed. Additional morphometric analyses were run in SPM12 software (see text footnote 2) in the MATLAB environment. SurfIce software was used to plot the brain maps.^3^

## Results

### Child Trauma Questionnaire

For BPD patients the MVPA returned a significant correlation with the subscale CTQ-Sexual abuse equal to 0.37, *p* = 0.04, the mean squared error (MSE): 2.50, *p* = 0.03, Normalized MSE: 0.62, *p* = 0.03. Areas showing a stronger contribution to the model are bilaterally the Caudate, the Heschl, the amygdala, the right supplementary area, the left putamen and right Rolandic operculum, various portions of the cerebellum (see Table 2 and Figure 1). The other subscales (Emotional neglect, physical neglect, emotional abuse, physical abuse) did not returned significant results (all *p* > 0.05).

**TABLE 2:** ROI weights and voxel sizes of the circuit predicting the CTQ-Sexual abuse for BPD.

Label	Significance (%)	Volume (Voxels)
*Caudate_L*	2.0336	2212
*Heschl_L*	1.6133	549
*Amygdala_L*	1.5737	487
*Supp_Motor_Area_R*	1.5166	5336
*Putamen_L*	1.5113	2255
*Heschl_R*	1.4855	513
*Rolandic_Oper_R*	1.4844	2946
*Cerebelum_7b_R*	1.4741	692
*Cerebelum_Crus2_L*	1.4177	4105
*Cerebelum_Crus2_R*	1.3772	3901
*Caudate_R*	1.3768	2330
*Calcarine_L*	1.3180	5182
*Frontal_Inf_Oper_R*	1.2756	2838
*Vermis_9*	1.2705	388
*Temporal_Mid_L*	1.2520	11409
*Postcentral_R*	1.2367	6986
*Hippocampus_L*	1.2251	2221
*Rolandic_Oper_L*	1.2123	2402
*Vermis_4_5*	1.1954	1489
*Paracentral_Lobule_R*	1.1864	1608
*Cuneus_L*	1.1582	3484
*Cerebelum_Crus1_R*	1.1441	4791
*Occipital_Mid_R*	1.1389	4649
*Temporal_Inf_R*	1.1280	7209
*Pallidum_L*	1.1107	637
*Amygdala_R*	1.1078	571
*Temporal_Inf_L*	1.1022	7081
*SupraMarginal_R*	1.0642	3768
*Angular_R*	1.0474	3628
*ParaHippocampal_L*	1.0473	2344
*Parietal_Inf_R*	1.0390	2671

*Only regions with at least 1% contribution to the model are reported.*

For HC subjects the MVPA returned a significant correlation with the subscale CTQ-Emotional neglect equal to 0.52, *p* = 0.009, MSE: 0.46, *p* = 0.01, Normalized MSE: 0.18, *p* = 0.01. Areas showing a stronger contribution to the model are several portions of the cerebellum, the precentral gyrus, some portions of the occipital and the medial and orbitofrontal parts of the frontal lobes (see Table 3). The other subscales (Sexual abuse, physical neglect, emotional abuse, physical abuse) did not return significant results (all *p* > 0.05).

**TABLE 3:** ROI weights and voxel sizes of the circuit predicting the CTQ-Emotional neglect for HC.

Label	Significance (%)	Volume (Voxels)
*Cerebelum_7b_R*	2.0262	692
*Vermis_9*	1.8854	388
*Cerebelum_9_L*	1.5372	1407
*Cerebelum_Crus2_L*	1.4105	4105
*Cerebelum_9_R*	1.4088	1320
*Cerebelum_Crus2_R*	1.3980	3901
*Heschl_L*	1.3388	549
*Cingulum_Mid_R*	1.3229	5244
*Cerebelum_Crus1_L*	1.3014	5334
*Precentral_R*	1.2832	6310
*Occipital_Inf_L*	1.2767	2264
*Cerebelum_8_R*	1.2642	2603
*Cerebelum_7b_L*	1.2502	863
*Cerebelum_4_5_L*	1.2483	2715
*Cingulum_Ant_R*	1.2218	3123
*Frontal_Mid_R*	1.1987	9213
*Frontal_Mid_Orb_R*	1.1778	1583
*Frontal_Inf_Tri_R*	1.1656	3654
*Occipital_Mid_R*	1.1603	4649
*Cerebelum_8_L*	1.1493	2619
*Vermis_3*	1.1471	522
*Frontal_Mid_L*	1.1403	11129
*Frontal_Inf_Orb_R*	1.1205	3635
*Temporal_Inf_L*	1.1099	7081
*Cerebelum_10_R*	1.1086	286
*Fusiform_R*	1.1074	5731
*Angular_L*	1.0897	2739
*Vermis_4_5*	1.0742	1489
*Paracentral_Lobule_L*	1.0715	2490
*Angular_R*	1.0657	3628
*SupraMarginal_L*	1.0509	2879
*Cerebelum_4_5_R*	1.0329	1938
*Parietal_Sup_L*	1.0182	4364
*Postcentral_R*	1.0155	6986
*Rectus_L*	1.0145	1780
*Temporal_Pole_Mid_R*	1.0123	1810
*Temporal_Sup_L*	1.0008	5312

*Only regions with at least 1% contribution to the model are reported.*

### Zanarini Rating Scale for Borderline Personality Disorder

**FIGURE 2:**
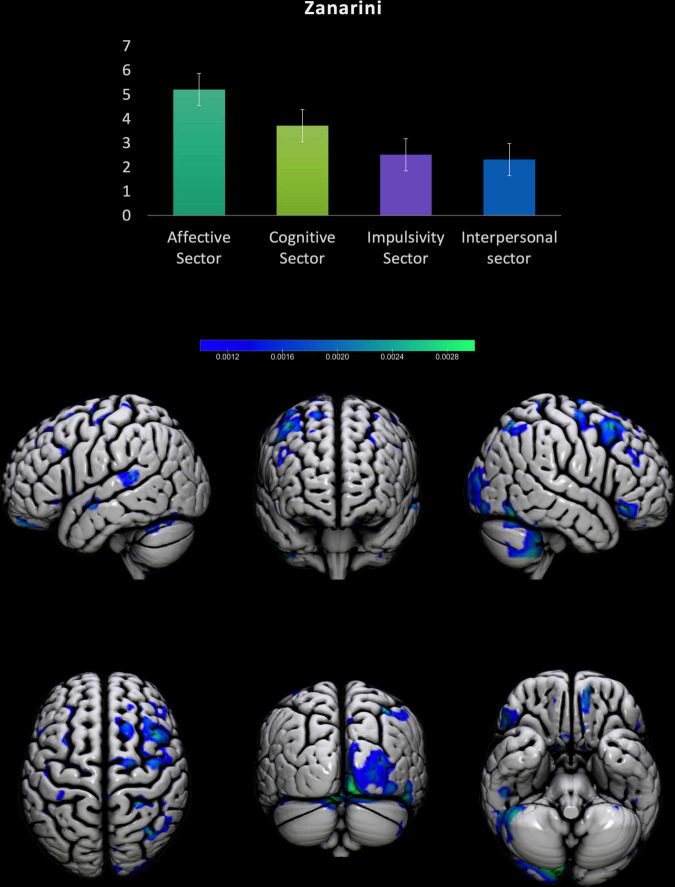
Results from the prediction of Zanarini scales for BPD patients. Upper part, results from the Zanarini sectors scores for BPD patients. Lower part, surface plots of the significant regions predicting Interpersonal problems subscale in BPD patients.

For BPD patients the MVPA returned a significant correlation with the subscale Zanarini Interpersonal problems, sector equal to 0.39, *p* = 0.04, MSE: 2.32, *p* = 0.04, Normalized MSE: 0.46, *p* = 0.04. Areas showing a stronger contribution to the model (see Table 4 and Figure 2). The other subscales (Affective sector, Impulsivity sector, Cognitive sector) did not returne significant results (all *p* > 0.05).

**TABLE 4:** ROI weights and voxel sizes of the circuit predicting the Zanarini-Interpersonal sector for BPD.

Labels	Significance (%)	Volume (Voxels)
*Temporal_Pole_Mid_R*	1.8963	1810
*Vermis_9*	1.7640	388
*Temporal_Inf_R*	1.7004	7209
*Frontal_Inf_Oper_R*	1.4985	2838
*Occipital_Inf_R*	1.3648	2411
*Cerebelum_Crus1_R*	1.3329	4791
*Angular_R*	1.3270	3628
*Cerebelum_7b_R*	1.3118	692
*Fusiform_R*	1.2755	5731
*ParaHippocampal_R*	1.2542	2557
*Cerebelum_Crus2_R*	1.1956	3901
*Frontal_Sup_R*	1.1873	8047
*Vermis_1_2*	1.1838	109
*Cerebelum_9_L*	1.1797	1407
*Occipital_Sup_R*	1.1739	3166
*Temporal_Mid_R*	1.1722	8803
*Parietal_Inf_R*	1.1675	2671
*Lingual_R*	1.1434	5574
*Cerebelum_9_R*	1.1147	1320
*Fusiform_L*	1.1135	5282
*SupraMarginal_L*	1.1046	2879
*Cingulum_Ant_L*	1.0933	3248
*Precuneus_R*	1.0850	7251
*Occipital_Mid_R*	1.0745	4649
*Frontal_Mid_R*	1.0669	9213
*Cingulum_Post_R*	1.0316	763
*Cerebelum_Crus1_L*	1.0220	5334
*SupraMarginal_R*	1.0189	3768

*Only regions with at least 1% contribution to the model are reported.*

### Additional Analyses

To understand the effect of diagnosis on the overall volumetric pattern, we also computed a simple Voxel-based morphometry. The contrast BPD > HC (FWE corrected) returned the following areas: right inferior occipital gyrus\right cerebellum, right supplementary motor cortex\right superior frontal gyrus, left superior frontal gyrus, right putamen\caudate, right supramarginal gyrus, right middle frontal gyrus, right orbito-frontal cortex, left middle temporal gyrus. The contrast HC > BPD (FWE corrected) returned the following areas: left post central gyrus, right precentral gyrus\superior frontal gyrus, right superior parietal lobe\precuneus.

## Discussion

The neural correlates of the Borderline Personality Disorder (BPD) clinical features are mostly unclear. So far, several neuroimaging studies have tried to unveil its neurofunctional and structural correlates, although using mass univariate approaches (see for instance: Herpertz et al., 2001; Völlm et al., 2004; Doell et al., 2020). To overcome previous methodological limitations, in the present study, we explored whether the main BPD features can be predicted by structural cerebral pattern by using a novel neuroimaging approach, combining clinical scales with multivariate pattern analysis (MVPA) based on Multiple Kernel Regression (MKR). More specifically, we explored the possibility that separate sets of areas would predict the main clinical features of BPD.

Evidence in the literature has shown that traumatic experiences are recognized as a risk factor for various psychiatric disorders (Widom et al., 2007; Chen et al., 2010), as well as the development of psychosis later in life (Thompson et al., 2014; Varese et al., 2012). Our results also corroborated this evidence on BPD patients, reinforcing the hypothesis that sexual abuse may be at the etiopathogenesis of the disorder (de Aquino Ferreira et al., 2018). Our results have shown that a complex cortico-subcortical set of areas predicted traumatic life events, such as the sexual abuse subscale, in BPD patients. Among all traumas, sexual trauma is the most common, severe and most associated with receiving a BPD diagnosis when adult. In line with previous studies, the set of areas predicting sexual abuse in BPD patients mainly involves subcortical regions such as the Caudate, Putamen and Amygdala (Herpertz et al., 2001; Xu et al., 2016). Morphometric alterations of striatum and putamen are associated with several neuropsychiatric disorders characterized by impulsive behavior, affect instability, and substance abuse (Luo et al., 2019; Lapomarda et al., 2021a,b). Notably, putamen is part of a cortical-striatal-thalamic circuit (Luo et al., 2019) that has been consistently implicated in affective processes of different psychiatric disorders (Fettes et al., 2017). In addition, the cerebellum, *via* connection with the basal ganglia and prefrontal cortex, is responsible for affective evaluation (Pierce and Péron, 2020; Piretti et al., 2021). Recent results have pointed out that the cerebellum may have a relevant role for emotions (Adamaszek et al., 2017; Pappaianni et al., 2018; Sorella et al., 2019; Lapomarda et al., 2021b). The contribution of the Heschl’s gyrus is also noticeable as it may increase function of posterior and anterior insula (Craig, 2005, 2009) in trauma-exposed patients contributing to multisensory dysfunctions in schizoaffective/schizophrenic patients (Quidé et al., 2017) psychotic patients (Aas et al., 2016) and is implicated in distorted internal dialoge in Eating disorders, and verbal hallucinations in schizophrenia. Furthermore, functional and volume abnormalities in amygdala and basal ganglia, for instance, has been suggested as the neural basis of the characterizing emotion dysregulation in BPD (Dadomo et al., 2016, 2018; Schulze et al., 2016; Grecucci et al., 2017; Frederickson et al., 2018; Grecucci et al., 2020). It is also interesting to note that the involvement of right lateralized set of cortical structure such as the right Rolandic Operculum, Paracentral Lobule and Inferior parietal regions (i.e., supramarginal and angular gyri) testimonies the bodily-related nature of the experienced trauma. Indeed, it has been hypothesized that these cortical areas would subserve the bodily-self-consciousness and altered emotional imitation (Grecucci et al., 2011; Salvato et al., 2020), which is typically altered in some psychiatric syndromes (Brugger and Lenggenhager, 2014).

The other subscales of the CTQ were not predicted by any other set of areas testifying that physical abuse, emotional abuse, physical neglect, and emotional neglect are not peculiar features of BPD. Interestingly, we found that a set of areas in the healthy brain predicted the CTQ subscale of emotional neglect. This finding provides evidence on the impact on the brain of specific relationship patterns in which the significant other disregarded, ignored, invalidated, or unappreciated individual’s affectional needs.

Lastly, our findings have shown that a specific set of regions predicts interpersonal problems in BPD patients. This evidence confirms the pivotal contribution of interpersonal problems in BPD, which are considered as the most characteristic and discriminative feature of the disorder (Fossati et al., 1999; Johansen et al., 2004; Gunderson, 2007). Patients affected by BPD frequently experience unstable and intense relationships with an alternation between idealization and devaluation (Lazarus et al., 2020). They also experience high interpersonal sensitivity and efforts to avoid abandonment (Domes et al., 2009; American Psychiatric Association [APA], 2013). In particular, the contribution the temporal lobe and cerebellar regions, involved in the set of predictor areas, are suggestive of such behavioral outcome in patients with BPD. Interpersonal skills (e.g., theory of mind) have been mostly associated with temporal pole activity in healthy and pathological subjects. Furthermore, temporal region have also been associated with social well-being (Gallagher and Frith, 2003; Giovagnoli et al., 2011; Kong et al., 2016). The cerebellar contribution to the prediction of this interpersonal behavioral problem in BPD confirms the role of this region in affective and interpersonal life. For instance, it has been demonstrated that lesion to “limbic cerebellum” (i.e., vermis) dysregulation of affect (Schmahmann et al., 2007). Moreover, the role of the cerebellum in social interaction has been highlighted (Leggio and Olivito, 2018). Notably, the majority of the areas found (with the exception of the left post central gyrus, the right precentral gyrus\superior frontal gyrus, and the right superior parietal lobe\precuneus) showed increased GM for BPD compared to HC, partially confiming, but also expanding previous Voxel-based morphometric analyses (see the review of Yu et al., 2019).

## Conclusion

Our work shows how combining clinical scales with multivariate pattern analysis (MVPA) based on Multiple Kernel Regression (MKR) provides important insights into which different aspects of BPD might link to different brain structures. Using two different specific instruments, to evaluate, respectively, the traumatic experiences lived during childhood and clinically relevant symptoms of borderline personality disorder we find a complex cortico-subcortical set of areas predict sexual trauma and interpersonal problems, that are the most common, severe and most associated symptoms in BPD. While further replication is warranted, due to the small sample size, our findings underscore the need to delve into structural brain patterns, not only based on symptom structure, but possibly also based on the persistent traumatic events inherent in many BPD patients. This study also contains some limitations. Firstly, the sample size is quite small for this kind of analyses. Future studies may want to extend and possibly replicate these findings. Unfortunately, the availability of pure BPD patients is not common as for other psychiatric disorders. Furthermore, healthy subjects did not perform the Zanarini scale. Future studies may overcome these issues. Last, but not least, these results may lead in the next future to new treatment possibilities. We hypothesize that neurostimulation protocols specifically focused on the circuit outlined in this study may help to ameliorate emotional disturbances displayed by BPD patients after sexual trauma.

## Data Availability Statement

Publicly available datasets were analyzed in this study. This data can be found here: OpenNeuro database, accession number ds000214.

## Ethics Statement

Ethical review and approval was not required for the study on human participants in accordance with the local legislation and institutional requirements. Written informed consent for participation was not required for this study in accordance with the national legislation and the institutional requirements.

## Author Contributions

HD: conceptualization, writing—original draft preparation, and writing—reviewing and editing. GS: writing—original draft preparation and writing—reviewing and editing. GL: preprocessing of MRI data and writing—reviewing and editing. ZC: writing—reviewing and editing. IM: conceptualization, writing—original draft preparation, and writing—reviewing and editing. AG: conceptualization, data curation, machine learning formal analysis, project management, and writing—reviewing and editing. All authors contributed to the article and approved the submitted version.

## Conflict of Interest

The authors declare that the research was conducted in the absence of any commercial or financial relationships that could be construed as a potential conflict of interest.

## Publisher’s Note

All claims expressed in this article are solely those of the authors and do not necessarily represent those of their affiliated organizations, or those of the publisher, the editors and the reviewers. Any product that may be evaluated in this article, or claim that may be made by its manufacturer, is not guaranteed or endorsed by the publisher.

## Footnotes

1
http://www.neuro.uni-jena.de/cat/

2
http://www.fil.ion.ucl.ac.uk/spm/software

3
https://www.nitrc.org/projects/surfice/

## References

[B1] Aas M. Henry C. Andreassen O. A. Bellivier F. Melle I. Etain B. (2016). The role of childhood trauma in bipolar disorders. *Int. J. Bipolar Disord.* 4:2. 10.1186/s40345-015-0042-0 26763504 PMC4712184

[B2] Adamaszek M. D’Agata F. Ferrucci R. Habas C. Keulen S. Kirkby K. C. (2017). Consensus paper: cerebellum and emotion. *Cerebellum* 16 552–576. 10.1007/s12311-016-0815-8 27485952

[B3] Aguilar-Ortiz S. Salgado-Pineda P. Marco-Pallarés J. Pascual J. C. Vega D. Soler J. (2018). Abnormalities in gray matter Vol. in patients with borderline personality disorder and their relation to lifetime depression: a VBM study. *PLoS One* 13:e0191946. 10.1371/journal.pone.0191946 29466364 PMC5842882

[B4] American Psychiatric Association [APA] (2013). *Diagnostic and Statistical Manual of Mental Disorders*, 5th Edn. Arlington, VA: American Psychiatric Publishing.

[B5] Ball J. S. Links P. S. (2009). Borderline personality disorder and childhood trauma: evidence for a causal relationship. *Curr. Psychiatry Rep.* 11 63–68. 10.1007/s11920-009-0010-4 19187711

[B6] Brugger P. Lenggenhager B. (2014). The bodily self and its disorders: neurological, psychological and social aspects. *Curr. Opin. Neurol.* 27 644–652. 10.1097/WCO.0000000000000151 25333602

[B7] Chen L. P. Murad M. H. Paras M. L. Colbenson K. M. Sattler A. L. Goranson E. N. (2010). Sexual abuse and lifetime diagnosis of psychiatric disorders: systematic review and meta-analysis. *Mayo Clin. Proc.* 85 618–629. 10.4065/mcp.2009.0583 20458101 PMC2894717

[B8] Ciocca G. Jannini T. B. Ribolsi M. Rossi R. Niolu C. Siracusano A. (2021). Sexuality in ultra-high risk for psychosis and first-episode psychosis. a systematic review of literature. *Front. Psychiatry* 12:750033. 10.3389/fpsyt.2021.750033 34777053 PMC8579023

[B9] Craig A. D. (2005). Forebrain emotional asymmetry: a neuroanatomical basis? *Trends Cogn. Sci.* 9 566–571. 10.1016/j.tics.2005.10.005 16275155

[B10] Craig A. D. (2009). How do you feel–now? the anterior insula and human awareness. *Nat. Rev. Neurosci.* 10 59–70. 10.1038/nrn2555 19096369

[B11] Dadomo H. Grecucci A. Giardini I. Ugolini E. Carmelita A. Panzeri M. (2016). Schema therapy for emotional dysregulation: theoretical implication and clinical applications. *Front. Psychol.* 7:1987. 10.3389/fpsyg.2016.01987 28066304 PMC5177643

[B12] Dadomo H. Panzeri M. Caponcello D. Carmelita A. Grecucci A. (2018). Schema therapy for emotional dysregulation in personality disorders: a review. *Curr. Opin. Psychiatry* 31 43–49. 10.1097/YCO.0000000000000380 29120915

[B13] Davies G. Hayward M. Evans S. Mason O. (2020). A systematic review of structural MRI investigations within borderline personality disorder: identification of key psychological variables of interest going forward. *Psychiatry Res.* 286:112864. 10.1016/j.psychres.2020.112864 32163818

[B14] de Aquino Ferreira L. F. Queiroz Pereira F. H. Neri Benevides A. Aguiar Melo M. C. (2018). Borderline personality disorder and sexual abuse: a systematic review. *Psychiatry Res.* 262 70–77. 10.1016/j.psychres.2018.01.043 29407572

[B15] De Panfilis C. Schito G. Generali I. Gozzi L. Ossola P. Marchesi C. (2019). Emotions at the border: increased punishment behavior during fair interpersonal exchanges in borderline personality disorder. *J. Abnorm. Psychol.* 128 162–172. 30714797 10.1037/abn0000404

[B16] Doell K. C. Olié E. Courtet P. Corradi-Dell’Acqua C. Perroud N. Schwartz S. (2020). Atypical processing of social anticipation and feedback in borderline personality disorder. *NeuroImage. Clin.* 25:102126. 31884223 10.1016/j.nicl.2019.102126 PMC6938803

[B17] Domes G. Schulze L. Herpertz S. C. (2009). Emotion recognition in borderline personality disorder-a review of the literature. *J. Personal. Disord.* 23 6–19. 10.1521/pedi.2009.23.1.6 19267658

[B18] Fettes P. Schulze L. Downar J. (2017). Cortico-striatal-thalamic loop circuits of the orbitofrontal cortex: promising therapeutic targets in psychiatric illness. *Front. Systems Neurosci.* 11:25. 10.3389/fnsys.2017.00025 28496402 PMC5406748

[B19] Fossati A. Maffei C. Bagnato M. Donati D. Namia C. Novella L. (1999). Latent structure analysis of DSM-IV borderline personality disorder criteria. *Compr. Psychiatry* 40 72–79. 10.1016/s0010-440x(99)90080-9 9924881

[B20] Frederickson J. J. Messina I. Grecucci A. (2018). Dysregulated anxiety and dysregulating defenses: toward an emotion regulation informed dynamic psychotherapy. *Front. Psychol.* 9:2054. 10.3389/fpsyg.2018.02054 30455650 PMC6230578

[B21] Gallagher H. L. Frith C. D. (2003). Functional imaging of ‘theory of mind’. *Trends Cogn. Sci.* 7 77–83. 10.1016/s1364-6613(02)00025-6 12584026

[B22] Giovagnoli A. R. Franceschetti S. Reati F. Parente A. Maccagnano C. Villani F. (2011). Theory of mind in frontal and temporal lobe epilepsy: cognitive and neural aspects. *Epilepsia* 52 1995–2002. 10.1111/j.1528-1167.2011.03215.x 21883176

[B23] Grecucci A. Job R. Frederickson J. (eds) (2017). Advances in emotion regulation: from neuroscience to psychotherapy. *Front. Psychol.* 8, 1–4. 10.3389/fpsyg.2017.00985 28680409 PMC5479113

[B24] Grecucci A. Koch I. Rumiati I. R. (2011). The role of emotional context in facilitating imitative actions. *Acta Psychol.* 138 311–315. 10.1016/j.actpsy.2011.07.005 21920488

[B25] Grecucci A. Messina I. Amodeo L. Lapomarda G. Crescentini C. Dadomo H. (2020). A dual route model for regulating emotions: comparing models, techniques and biological mechanisms. *Front. Psychol.* 11:930. 10.3389/fpsyg.2020.00930 32581903 PMC7287186

[B26] Grecucci A. Pappaianni E. Siugzdaite R. Thneuick A. Job R. (2015). Mindful emotion regulation: psychological and neural mechanisms. *BioMed Res.* 2015:670724. 10.1155/2015/670724 PMC4475519 26137490

[B27] Grecucci A. Rubicondo D. Siugzdaite R. Surian L. Job R. (2016). Uncovering the social deficits in the autistic brain. a source-based morphometric study. *Front. Neurosci.* 10:388. 10.3389/fnins.2016.00388 27630538 PMC5005369

[B28] Gunderson J. G. (2007). Disturbed relationships as a phenotype for borderline personality disorder. *Am. J. Psychiatry* 164 1637–1640. 10.1176/appi.ajp.2007.07071125 17974925

[B29] Herpertz S. C. Dietrich T. M. Wenning B. Krings T. Erberich S. G. Willmes K. (2001). Evidence of abnormal amygdala functioning in borderline personality disorder: a functional MRI study. *Biol. Psychiatry* 50 292–298. 10.1016/s0006-3223(01)01075-7 11522264

[B30] Johansen M. Karterud S. Pedersen G. Gude T. Falkum E. (2004). An investigation of the prototype validity of the borderline DSM-IV construct. *Acta Psychiatr. Scand.* 109 289–298. 10.1046/j.1600-0447.2003.00268.x 15008803

[B31] Johnson J. G. Cohen P. Brown J. Smailes E. M. Bernstein D. P. (1999). Childhood maltreatment increases risk for personality disorders during early adulthood. *Arch. Gen. Psychiatry* 56 600–606. 10.1001/archpsyc.56.7.600 10401504

[B32] Kim H. J. Kim J. E. Lee S. H. (2021). Early trauma is associated with poor pharmacological treatment response in patients with panic disorder. *Psychiatry Invest.* 18:249. 10.30773/pi.2020.0380 33735547 PMC8016688

[B33] Koenigsberg H. W. Harvey P. D. Mitropoulou V. Schmeidler J. New A. S. Goodman M. (2002). Characterizing affective instability in borderline personality disorder. *Am. J. Psychiatry* 159 784–788. 10.1176/appi.ajp.159.5.784 11986132

[B34] Kong F. Xue S. Wang X. (2016). Amplitude of low frequency fluctuations during resting state predicts social well-being. *Biol. Psychol.* 118 161–168. 10.1016/j.biopsycho.2016.05.012 27263835

[B35] Lapomarda G. Grecucci A. Messina I. Pappaianni E. Dadomo H. (2021a). Common and different gray and white matter alterations in bipolar and borderline personality disorder. *Brain Res.* 1762:147401. 10.1016/j.brainres.2021.147401 33675742

[B36] Lapomarda G. Pappaianni E. Siugzdaite R. Sanfey A. G. Rumiati R. I. Grecucci A. (2021b). Out of control: an altered parieto-occipital-cerebellar network for impulsivity in bipolar disorder. *Behav. Brain Res.* 406:113228. 33684426 10.1016/j.bbr.2021.113228

[B37] Lazarus S. A. Beeney J. E. Howard K. P. Strunk D. R. Pilkonis P. Cheavens J. S. (2020). Characterization of relationship instability in women with borderline personality disorder: a social network analysis. *Personal. Disord. Theory Res. Treatment* 11:312. 10.1037/per0000380 31804129

[B38] Leggio M. Olivito G. (2018). Topography of the cerebellum in relation to social brain regions and emotions. *Handb. Clin. Neurol.* 154 71–84. 10.1016/B978-0-444-63956-1.00005-9 29903453

[B39] Lenzenweger M. F. Lane M. C. Loranger A. W. Kessler R. C. (2007). DSM-IV personality disorders in the national comorbidity survey replication. *Biol. Psychiatry* 62 553–564. 10.1016/j.biopsych.2006.09.019 17217923 PMC2044500

[B40] Lieb K. Zanarini M. C. Schmahl C. Linehan M. M. Bohus M. (2004). Borderline personality disorder. *Lancet (London, England)* 364 453–461. 10.1016/S0140-6736(04)16770-6 15288745

[B41] Lobbestael J. Arntz A. (2010). Emotional, cognitive and physiological correlates of abuse-related stress in borderline and antisocial personality disorder. *Behav. Res. Ther.* 48 116–124. 10.1016/j.brat.2009.09.015 19854433

[B42] Lobbestael J. Arntz A. Bernstein D. P. (2010). Disentangling the relationship between different types of childhood maltreatment and personality disorders. *J. Personal. Disord.* 24 285–295. 10.1521/pedi.2010.24.3.285 20545495

[B43] Luo X. Mao Q. Shi J. Wang X. Li C. R. (2019). Putamen gray matter volumes in neuropsychiatric and neurodegenerative disorders. *World J. Psychiatry Mental Health Res.* 3:1020. PMC6641567 31328186

[B44] McGlashan T. H. Grilo C. M. Skodol A. E. Gunderson J. G. Shea M. T. Morey L. C. (2000). The collaborative longitudinal personality disorders study: baseline Axis I/II and II/II diagnostic co-occurrence. *Acta Psychiatr. Scand.* 102 256–264. 10.1034/j.1600-0447.2000.102004256.x 11089725

[B45] Minzenberg M. J. Fan J. New A. S. Tang C. Y. Siever L. J. (2008). Frontolimbic structural changes in borderline personality disorder. *J. Psychiatr. Res.* 42 727–733. 10.1016/j.jpsychires.2007.07.015 17825840 PMC2708084

[B46] Mortensen J. A. Rasmussen L. A. Håberg A. (2010). Trait impulsivity in female patients with borderline personality disorder and matched controls. *Acta Neuropsychiatrica* 22 139–149. 10.1111/j.1601-5215.2010.00468.x 26952804

[B47] Mourao-Miranda J. Reinders A. A. Rocha-Rego V. Lappin J. Rondina J. Morgan C. (2012). Individualized prediction of illness course at the first psychotic episode: a support vector machine MRI study. *Psychol. Med.* 42 1037–1047. 10.1017/S0033291711002005 22059690 PMC3315786

[B48] Müller M. Ajdacic-Gross V. Rodgers S. Kleim B. Seifritz E. Vetter S. (2018). Predictors of remission from PTSD symptoms after sexual and non-sexual trauma in the community: a mediated survival-analytic approach. *Psychiatry Res.* 260 262–271. 10.1016/j.psychres.2017.11.068 29220684

[B49] Norman K. A. Polyn S. M. Detre G. J. Haxby J. V. (2006). Beyond mind-reading: multi-voxel pattern analysis of fMRI data. *Trends Cogn. Sci.* 10 424–430. 10.1016/j.tics.2006.07.005 16899397

[B50] Nunes P. M. Wenzel A. Borges K. T. Porto C. R. Caminha R. M. de Oliveira I. R. (2009). Volumes of the hippocampus and amygdala in patients with borderline personality disorder: a meta-analysis. *J. Personal. Disord.* 23 333–345. 10.1521/pedi.2009.23.4.333 19663654

[B51] Orrù G. Pettersson-Yeo W. Marquand A. F. Sartori G. Mechelli A. (2012). Using support vector machine to identify imaging biomarkers of neurological and psychiatric disease: a critical review. *Neurosci. Biobehav. Rev.* 36 1140–1152. 10.1016/j.neubiorev.2012.01.004 22305994

[B52] Pappaianni E. De Pisapia N. Siugzdaite R. Crescentini C. Calcagnì A. Job R. (2020). Less is more: morphometric and psychological differences between low and high reappraisers. *Cogn. Behav. Neurosci.* 20 128–140. 10.3758/s13415-019-00757-5 31858436 PMC7613187

[B53] Pappaianni E. Siugzdaite R. Vettori S. Venuti P. Job R. Grecucci A. (2018). Three shades of grey: detecting brain abnormalities in children with autism using source-, voxel- and surface-based morphometry. *Eur. J. Neurosci.* 47 690–700. 10.1111/ejn.13704 28921735

[B54] Paris J. Zweig-Frank H. Guzder J. (1994). Risk factors for borderline personality in male outpatients. *J. Nerv. Ment. Dis.* 182 375–380. 10.1097/00005053-199407000-00002 8021636

[B55] Pierce J. E. Péron J. (2020). The basal ganglia and the cerebellum in human emotion. *Soc. Cogn. Affect. Neurosci.* 15 599–613. 10.1093/scan/nsaa076 32507876 PMC7328022

[B56] Piretti L. Pappaianni E. Lunardelli A. Zorzenon I. Ukmar M. Pesavento V. (2020). The role of amygdala in self-conscious emotions in a patient with acquired bilateral damage. *Front. Neurosci.* 14:677. 10.3389/fnins.2020.00677 32733192 PMC7360725

[B57] Piretti L. Pappaianni E. Rumiati R. Job R. Grecucci A. (2021). Dissociating the role of dlPFC and dACC/dmPFC in emotional processing using tDCS. *Cogn. Affect. Behav. Neurosci.* 7, 1–11. 10.3758/s13415-021-00952-3 34676495

[B58] Poldrack R. A. Gorgolewski K. J. (2017). OpenfMRI: open sharing of task fMRI data. *NeuroImage* 144 259–261. 10.1016/j.neuroimage.2015.05.073 26048618 PMC4669234

[B59] Poppa T. Droutman V. Amaro H. Black D. Arnaudova I. Monterosso J. (2019). Sexual trauma history is associated with reduced orbitofrontal network strength in substance-dependent women. *Neuroimage: Clin.* 24:101973. 10.1016/j.nicl.2019.101973 31472330 PMC6728879

[B60] Preißler S. Dziobek I. Ritter K. Heekeren H. R. Roepke S. (2010). Social cognition in borderline personality disorder: evidence for disturbed recognition of the emotions, thoughts, and intentions of others. *Front. Behav. Neurosci.* 4:182. 10.3389/fnbeh.2010.00182 21151817 PMC2999836

[B61] Quidé Y. O’Reilly N. Rowland J. E. Carr V. J. Elzinga B. M. Green M. J. (2017). Effects of childhood trauma on working memory in affective and non-affective psychotic disorders. *Brain Imag. Behav.* 11 722–735. 10.1007/s11682-016-9548-z 27090803

[B62] Ruocco A. C. Amirthavasagam S. Zakzanis K. K. (2012). Amygdala and hippocampal volume reductions as candidate endophenotypes for borderline personality disorder: a meta-analysis of magnetic resonance imaging studies. *Psychiatry Res.* 201 245–252. 10.1016/j.pscychresns.2012.02.012 22507760

[B63] Ruocco A. C. Rodrigo A. H. McMain S. F. Page-Gould E. Ayaz H. Links P. S. (2016). Predicting treatment outcomes from prefrontal cortex activation for self-harming patients with borderline personality disorder: a preliminary study. *Front. Hum. Neurosci.* 10:220. 10.3389/fnhum.2016.00220 27242484 PMC4870399

[B64] Salvato G. Richter F. Sedeño L. Bottini G. Paulesu E. (2020). Building the bodily self-awareness: evidence for the convergence between interoceptive and exteroceptive information in a multilevel kernel density analysis study. *Hum. Brain Mapp.* 41 401–418. 10.1002/hbm.24810 31609042 PMC7268061

[B65] Saviola F. Pappaianni E. Monti A. Grecucci A. Jovicich J. De Pisapia N. (2020). Trait and state anxiety are mapped differently in the human brain. *Sci. Rep.* 10:11112. 32632158 10.1038/s41598-020-68008-z PMC7338355

[B66] Schmahmann J. D. Weilburg J. B. Sherman J. C. (2007). The neuropsychiatry of the cerebellum - insights from the clinic. *Cerebellum (London, England)* 6 254–267. 10.1080/14734220701490995 17786822

[B67] Schrouff J. Monteiro J. M. Portugal L. Rosa M. J. Phillips C. Mourão-Miranda J. (2018). Embedding anatomical or functional knowledge in whole-brain multiple kernel learning models. *Neuroinformatics* 16 117–143. 10.1007/s12021-017-9347-8 29297140 PMC5797202

[B68] Schrouff J. Monteiro J. Joao Rosa M. Portugal L. Phillips C. Mourao-Miranda J. (2014). Can we interpret linear kernel machine learning models using anatomically labelled regions? *Personal Commun.*

[B69] Schrouff J. Rosa M. J. Rondina J. M. Marquand A. F. Chu C. Ashburner J. (2013). PRoNTo: pattern recognition for neuroimaging toolbox. *Neuroinformatics* 11 319–337. 10.1007/s12021-013-9178-1 23417655 PMC3722452

[B70] Schulze L. Schmahl C. Niedtfeld I. (2016). Neural correlates of disturbed emotion processing in borderline personality disorder: a multimodal meta-analysis. *Biol. Psychiatry* 79 97–106. 10.1016/j.biopsych.2015.03.027 25935068

[B71] Shearer S. L. Peters C. P. Quaytman M. S. Ogden R. L. (1990). Frequency and correlates of childhood sexual and physical abuse histories in adult female borderline inpatients. *Am. J. Psychiatry* 147 214–216. 10.1176/ajp.147.2.214 2301663

[B72] Sorella S. Lapomarda G. Messina I. Frederickson J. J. Siugzdaite R. Job R. (2019). Testing the expanded continuum hypothesis of schizophrenia and bipolar disorder. Neural and psychological evidence for shared and distinct mechanisms. *NeuroImage. Clin.* 23:101854. 10.1016/j.nicl.2019.101854 31121524 PMC6529770

[B73] Stanley B. Perez-Rodriguez M. M. Labouliere C. Roose S. (2018). A neuroscience-oriented research approach to borderline personality disorder. *J. Personal. Disord.* 10.1521/pedi_2018_32_326 Online ahead of print 29469663

[B74] Stiglmayr C. E. Shapiro D. A. Stieglitz R. D. Limberger M. F. Bohus M. (2001). Experience of aversive tension and dissociation in female patients with borderline personality disorder – a controlled study. *J. Psychiatr. Res.* 35 111–118. 10.1016/s0022-3956(01)00012-7 11377440

[B75] Takamiya A. Kishimoto T. Hirano J. Nishikata S. Sawada K. Kurokawa S. (2020). Neuronal network mechanisms associated with depressive symptom improvement following electroconvulsive therapy. *Psychol. Med.* 10.1017/S0033291720001518 Online ahead of print 32476629 PMC8640363

[B76] Thompson A. D. Nelson B. Yuen H. P. Lin A. Amminger G. P. McGorry P. D. (2014). Sexual trauma increases the risk of developing psychosis in an ultra high-risk “prodromal” population. *Schizophrenia Bull.* 40 697–706. 10.1093/schbul/sbt032 23455040 PMC3984502

[B77] Trull T. J. Jahng S. Tomko R. L. Wood P. K. Sher K. J. (2010). Revised NESARC personality disorder diagnoses: gender, prevalence, and comorbidity with substance dependence disorders. *J. Personal. Disord.* 24 412–426. 10.1521/pedi.2010.24.4.412 20695803 PMC3771514

[B78] Tzourio-Mazoyer N. Landeau B. Papathanassiou D. Crivello F. Etard O. Delcroix N. (2002). Automated anatomical labeling of activations in SPM using a macroscopic anatomical parcellation of the MNI MRI single-subject brain. *NeuroImage* 15 273–289. 10.1006/nimg.2001.0978 11771995

[B79] Varese F. Smeets F. Drukker M. Lieverse R. Lataster T. Viechtbauer W. (2012). Childhood adversities increase the risk of psychosis: a meta-analysis of patient-control, prospective- and cross-sectional cohort studies. *Schizophrenia Bull.* 38 661–671. 10.1093/schbul/sbs050 22461484 PMC3406538

[B80] Völlm B. Richardson P. Stirling J. Elliott R. Dolan M. Chaudhry I. (2004). Neurobiological substrates of antisocial and borderline personality disorder: preliminary results of a functional fMRI study. *Criminal Behav. Mental Health: CBMH* 14 39–54. 10.1002/cbm.559 14654860

[B81] Whitfield-Gabrieli S. Ghosh S. S. Nieto-Castanon A. Saygin Z. Doehrmann O. Chai X. J. (2016). Brain connectomics predict response to treatment in social anxiety disorder. *Mol. Psychiatry* 21 680–685. 10.1038/mp.2015.109 26260493

[B82] Widom C. S. DuMont K. Czaja S. J. (2007). A prospective investigation of major depressive disorder and comorbidity in abused and neglected children grown up. *Arch. Gen. Psychiatry* 64 49–56. 10.1001/archpsyc.64.1.49 17199054

[B83] Xu T. Cullen K. R. Mueller B. Schreiner M. W. Lim K. O. Schulz S. C. (2016). Network analysis of functional brain connectivity in borderline personality disorder using resting-state fMRI. *NeuroImage: Clin.* 11 302–315. 10.1016/j.nicl.2016.02.006 26977400 PMC4782004

[B84] Yassa M. A. Stark C. E. (2009). A quantitative evaluation of cross-participant registration techniques for MRI studies of the medial temporal lobe. *NeuroImage* 44 319–327. 10.1016/j.neuroimage.2008.09.016 18929669

[B85] Yu H. Meng Y. J. Li X. J. Zhang C. Liang S. Li M. L. (2019). Common and distinct patterns of grey matter alterations in borderline personality disorder and bipolar disorder: voxel-based meta-analysis. *Br. J. Psychiatry* 215 395–403. 10.1192/bjp.2019.44 30846010

[B86] Zanarini M. C. Frankenburg F. R. DeLuca C. J. Hennen J. Khera G. S. Gunderson J. G. (1998). The pain of being borderline: dysphoric states specific to borderline personality disorder. *Harv. Rev. Psychiatry* 6 201–207. 10.3109/10673229809000330 10370445

[B87] Zanarini M. C. Gunderson J. G. Frankenburg F. R. (1990). Cognitive features of borderline personality disorder. *Am. J. Psychiatry* 147 57–63. 10.1176/ajp.147.1.57 2293789

[B88] Zanarini M. C. Gunderson J. G. Marino M. F. Schwartz E. O. Frankenburg F. R. (1989). Childhood experiences of borderline patients. *Compr. Psychiatry* 30 18–25. 10.1016/0010-440x(89)90114-4 2924564

[B89] Zanarini M. C. Yong L. Frankenburg F. R. Hennen J. Reich D. B. Marino M. F. (2002). Severity of reported childhood sexual abuse and its relationship to severity of borderline psychopathology and psychosocial impairment among borderline inpatients. *J. Nerv. Ment. Dis.* 190 381–387. 10.1097/00005053-200206000-00006 12080208

[B90] Zhang Y. Liu F. Chen H. Li M. Duan X. Xie B. (2015). Intranetwork and internetwork functional connectivity alterations in post-traumatic stress disorder. *J. Affect. Disord.* 187 114–121. 10.1016/j.jad.2015.08.043 26331685

[B91] Zimmerman M. Rothschild L. Chelminski I. (2005). The prevalence of DSM-IV personality disorders in psychiatric outpatients. *Am. J. Psychiatry* 162 1911–1918. 10.1176/appi.ajp.162.10.1911 16199838

[B92] Zlotnick C. Johnson D. M. Yen S. Battle C. L. Sanislow C. A. Skodol A. E. (2003). Clinical features and impairment in women with Borderline Personality Disorder (BPD) with Posttraumatic Stress Disorder. (PTSD), BPD Without PTSD, and Other- Personality Disorders with PTSD. *J. Nervous Mental Dis.* 191 706–713. 10.1097/01.nmd.0000095122.29476.ff 14614337

